# Synthesis and Characterization of Oxide Chloride Sr_2_VO_3_Cl, a Layered *S* = 1 Compound

**DOI:** 10.1021/acsomega.3c01151

**Published:** 2023-04-05

**Authors:** Johnny A. Sannes, Ranjith K. Kizhake Malayil, Laura T. Corredor, Anja U. B. Wolter, Hans-Joachim Grafe, Martin Valldor

**Affiliations:** † Department of Chemistry, University of Oslo, Sem Sælands vei 26, N-0371 Oslo, Norway; ‡ Leibniz Institute for Solid State Research, IFW Dresden, Helmholtzstraβe 20, 01069 Dresden, Germany

## Abstract

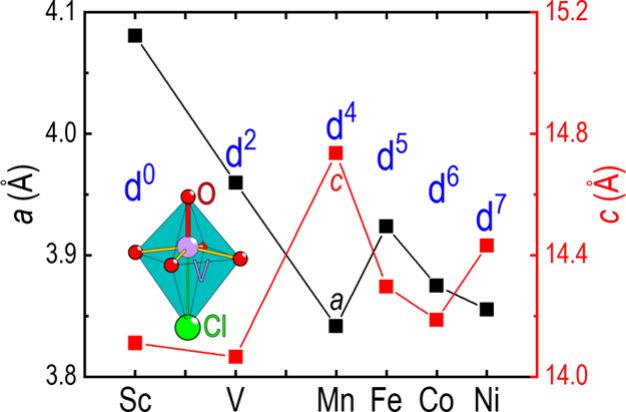

The mixed-anion compound with composition Sr_2_VO_3_Cl has been synthesized for the first time, using the conventional high-temperature solid-state synthesis technique in a closed silica ampule under inert conditions. This compound belongs to the known Sr_2_*Tm*O_3_Cl (*Tm* = Sc, Mn, Fe, Co, Ni) family, but with *Tm* = V. All homologues within this family can be described with the tetragonal space group *P*4/*nmm* (No. 129); from a Rietveld refinement of powder X-ray diffraction data on the *Tm* = V homologue, the unit cell parameters were determined to *a* = 3.95974(8) and *c* = 14.0660(4) Å, and the atomic parameters in the crystal structure could be estimated. The synthesized powder is black, implying that the compound is a semiconductor. The magnetic investigations suggest that Sr_2_VO_3_Cl is a paramagnet at high temperatures, exhibiting a μ_eff_ = 2.0 μ_B_ V^–1^ and antiferromagnetic (AFM) interactions between the magnetic vanadium spins (θ_CW_ = −50 K), in line with the V–O–V advantageous super-exchange paths in the V–O layers. Specific heat capacity studies indicate two small anomalies around 5 and 35 K, which however are not associated with long-range magnetic ordering. ^35^Cl ss-NMR investigations suggest a slow spin freezing below 4.2 K resulting in a glassy-like spin ground state.

## Introduction

Two-dimensional (2d) layered compounds are of interest for possible intercalation^1^ and superconductivity^2^ applications. A recent chemical design to increase the structural anisotropy, to form layered materials, is that of mixed-anion compounds.^3,4^ By using two different anions, even further effects can be obtained, such as polar, heteroleptic coordinations, and charge ordering. One of the most investigated 2d lattices are the Ruddlesden–Popper (RP) phases that usually are pure oxides. By introducing a part chlorine in the oxide lattice, its anisotropy should be altered. This was successful for the compound series Sr_2_*Tm*O_3_Cl (*Tm* = Sc,^5^ Mn,^6^ Fe,^7^ Co,^8^ Ni^9^), which is structurally related to the 214 RP phases. There are higher homologues of the oxychloride RP phases, such as, Sr_3_Co_2_O_5_Cl_2_ and Sr_4_Mn_3_O_8_Cl_2_, indicating the further design of RP phases if a further *Tm* can be introduced.^6,8^ Yet, a few of the *Tm*s in Sr_2_*Tm*O_3_Cl have not been reported. Herein, we describe the synthesis and fundamental properties of the vanadium homologue, Sr_2_VO_3_Cl. Note, IUPAC recommends to write Sr_2_VClO_3_, because of the alphabetical order of anions, but, for historical reasons, the halogen will be placed last in the formula throughout this report.

## Materials and Methods

In order to obtain an X-ray pure sample of the title compound, SrO, SrCl_2_ (Alfa Aesar 99.995%), V (Alfa Aesar 99.5%), and V_2_O_5_ (British Drug Houses, *pro analysis*) in amounts corresponding to the stoichiometry Sr_2_VO_2.875_Cl were used. SrO was made in-house from SrCO_3_, (Fluka ≥ 98%) by an over-night heating at 1030 °C under dynamic vacuum (*p* < 10^–2^ mbar). The obtained SrO was proven to be X-ray pure, and it was only handled without exposure to air. The slight oxygen non-stoichiometry was intentional, because all starting composition with more oxygen resulted in samples with small amounts of V(IV) compounds, such as SrVO_3_.^10^ Minor oxide impurities in the vanadium metal is expected to be the cause of this observation. The powder mixture was ground thoroughly in an agate mortar inside a glovebox (O_2_ and H_2_O < 2 ppm) and all handling of the precursors was performed therein. The powder mixture was placed in a corundum crucible, which was melt-sealed inside a silica ampule. The solid-state reaction was performed in a muffle furnace at 1100 ^°^C for 10 h (heating rate: 5° min^–1^) and the subsequent cooling was done at an ambient rate. Note that no reaction was observed between the sample and crucible after the solid-state reaction.

### Powder X-ray Diffraction (pXRD)

X-ray diffraction at room temperature was performed using a Bruker D8 Discover with a Bragg–Brentano geometry, a Ge(1 1 1) Johanssen monochromator to select CuKα_1_ radiation (λ = 1.5406 Å), and a Lynxeye as the detector. The obtained data were indexed using the software Topas.^11,12^ Further analyses, including Rietveld refinement, were done using the Jana2006 software package.^13^ In the refinement, the background was fitted by Chebyshev polynomials including 15 terms. First, the two unit cell parameters were refined together with the zero-position in a Le Bail fit, where the Pseudo-Voigt function was used to simulate the peak shapes. In the final refinement, all atomic fractional coordinates, as allowed by symmetry restrictions, as well as isotropic thermal displacement parameters were refined. Expecting anisotropic crystal morphology, preferred orientation along the (1 1 0) direction, according to March–Dollase, was included, significantly improving the overall fit. Within the diffraction range, there were 46 observed intensities that were simulated with 13 free parameters. All specific data are shown in Table 1.

**Table 1:** Result of the Rietveld Refinement

chemical formula	Sr_2_VO_3_Cl
fw (g mol^–1^)	309.63
temperature	ambient
λ (Å)	1.5406
crystal system	tetragonal
space group	*P*4/*nmm* (No. 129)
*a* (Å)	3.95974(8)
*c* (Å)	14.0660(4)
*V* (Å^3^)	220.549(8)
*Z*	2
GOF	0.14
*R_P_* (%)	5.66
*R_wp_* (%)	7.64
diff Fourier peak/hole (e Å^–3^)	0.35/–0.33
atom, Wyckoff, *x*, *y*, *z*, *U*_iso_	Sr1, 2*c*, 0.5, 0, 0.0965(2), 0.026(2)
	Sr2, 2*c*, 0, 0.5, −0.3464(2), 0.021(1)
	V1, 2*c*, 0.5, 0, −0.2138(4), 0.026(2)
	Cl1, 2*c*, 0, 0.5, 0.4227(5), 0.032(3)
	O1, 2*c*, 0.5, 0, −0.084(1), 0.034(6)
	O2, 4*f*, 0.5, 0.5, −0.2365(7), 0.032(4)
CSD number	2094715

### Scanning Electron Microscopy (SEM)

SEM images were captured using a Hitachi SU8230 field emission scanning electron microscope (FESEM). A current of 5 μA at an acceleration voltage of 5 kV was used. The elemental composition was determined using a XFlash 6|10 EDX detector. Averaged values of measurements from 10 different crystallites were used to estimate the elemental composition of the sample.

### Magnetic and Thermodynamic Measurements

A Quantum Design Physical Property Measurement System (PPMS) was used to measure the magnetic susceptibility at three different magnetic field strengths (μ_0_*H* = 0.1, 0.5, 1 T) from 4 to 375 K. The polycrystalline sample was packed in a polypropylene sample holder and measured in a VSM insert at 40 Hz with 2 mm amplitude. The specific heat capacity was studied from 2 to 300 K on a non-sintered pellet at zero magnetic field in the same PPMS using the standard non-adiabatic thermal relaxation method, including an addenda subtraction from the absolute signal.

### Solid-State Nuclear Magnetic Resonance (ss-NMR)

NMR measurements were performed using a Tecmag Apollo spectrometer with a standard probe head and a sweepable Oxford Instruments magnet. Temperatures were controlled using a ^4^He temperature insert (VTI). Temperatures below 4.2 K were achieved by pumping at the VTI. ^35^Cl NMR spectra were obtained using the frequency sweep method at a fixed magnetic field of 7 T.

## Results

The resulting powder of Sr_2_VO_3_Cl is completely black, and no larger crystals could be observed after synthesis. During characterization, it was observed that the sample is slightly sensitive in air/to humidity, slowly decomposing with time, which leads to visible changes in its magnetic properties.

### SEM/EDX

**Figure 1:**
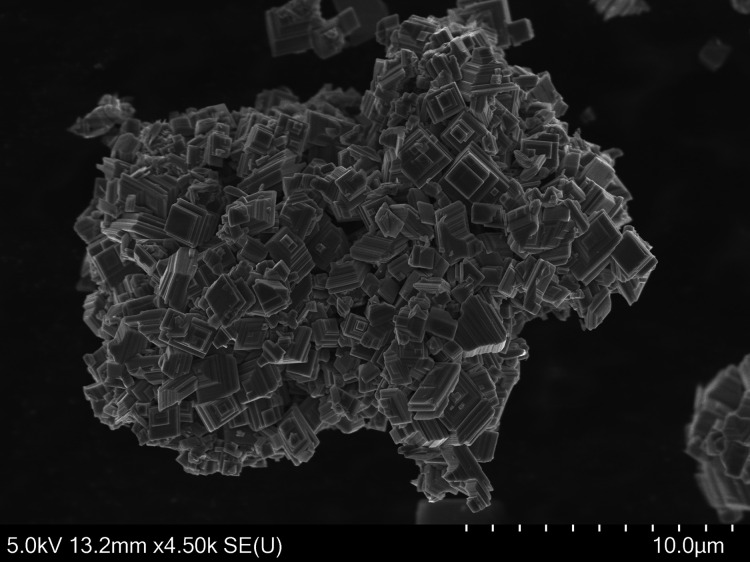
Scanning electron microscopy image of Sr_2_VO_3_Cl crystallites. A scale is added for size comparison. The total scale corresponds to 10 μm.

Observation done in SEM shows that the sample has a high degree of crystallinity, see Figure 1. The crystal morphology resembles stacked plates with angles of 90°. The semiquantitative elemental analysis, as determined by EDX on 10 different crystallites, is Sr_2.1(1)_V_0.94(5)_O_*x*_Cl_1.0(2)_ when assuming all elements but oxygen to add up to four. Despite the facts that the compound is a poor conductor and the polycrystalline nature of the sample can cause topology issues for the EDX detector, these results agree fairly well with the expectations.

### Powder X-ray Diffraction and Rietveld Refinement

**Figure 2:**
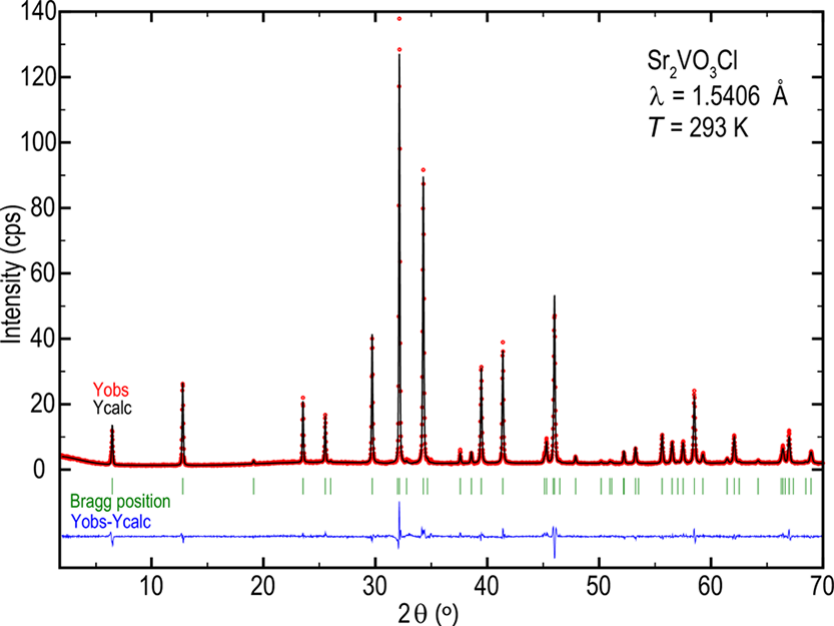
X-ray diffraction data of Sr_2_VO_3_Cl at room temperature (open red circles, *Y*_obs_). The Rietveld refinement (black line, *Y*_calc_) is superimposed, and the difference between the data and refinement is shown by the blue line (*Y*_obs_ – *Y*_calc_). The expected Bragg positions are indicated by green vertical lines. The asterisk marks an additional intensity from a secondary phase.

The synthesized sample exhibits high crystallinity: the peak shapes are all narrow and high intensities, even at high angles, are observed. When comparing the Rietveld refinement with the observed data, the sample seems to be almost X-ray pure. The largest deviation between the observed and calculated intensities occurs at the peaks of highest intensities, indicating minor errors in the peak-shape function. The largest extrinsic intensity (from a secondary phase) is found close to 2θ = 35°, as indicated by an asterisk in Figure 2. However, its origin is unclear, although a SrO reflection can be expected at that angle.

**Figure 3:**
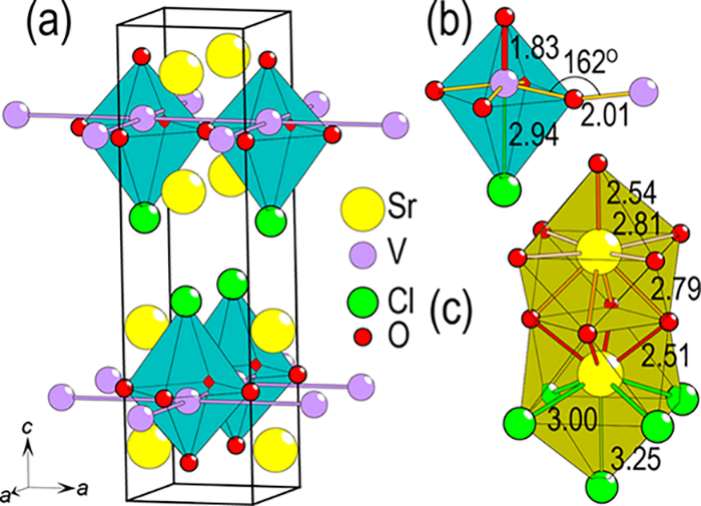
(a) Unit cell of Sr_2_VO_3_Cl highlighting the layered nature of the structure. (b) Vanadium atoms are coordinated to five oxygen and one chlorine in a 5 + 1 octahedral coordination. The V–O–V bond distances and angle are indicated. (c) Two differently coordinated strontium ions, where identical interatomic distances are indicated with equally colored lines. All given interatomic distances are in Å.

In the title compound, two different vanadium-to-oxygen distances of 1.83 and 2.01 Å are observed. These values are significant shorter compared to the V–O distances in V_2_O_3_ of 1.97 and 2.05 Å.^14^ The observed distance between vanadium and chlorine is 2.94 Å for Sr_2_VO_3_Cl, much larger than the observed bond length of 2.417 and 2.418 Å for VCl_3_.^15^ The calculated bond valence sum (BVS) for vanadium is 2.87 when including chlorine^16^ (and 2.73 without), close to the expected value 3. This suggests that the vanadium coordination is most suitably described as a distorted octahedron with a 5 + 1 coordination. The vanadium atoms form 2d-layers as indicated in Figure 3a. This structure is directly related to a 214 RP phase, with the main difference that one of the oxygen atoms has been replaced by a chlorine. The two strontium atoms are differently coordinated: one of the strontium atoms only coordinates to oxygen while the other coordinates to both oxygen and chlorine, see Figure 3c. From the observed V–O–V angle of 162°, magnetic super-exchange is expected to be important, probably giving rise to AFM interactions.^17,18^

### Magnetic and Specific Heat Measurements

**Figure 4:**
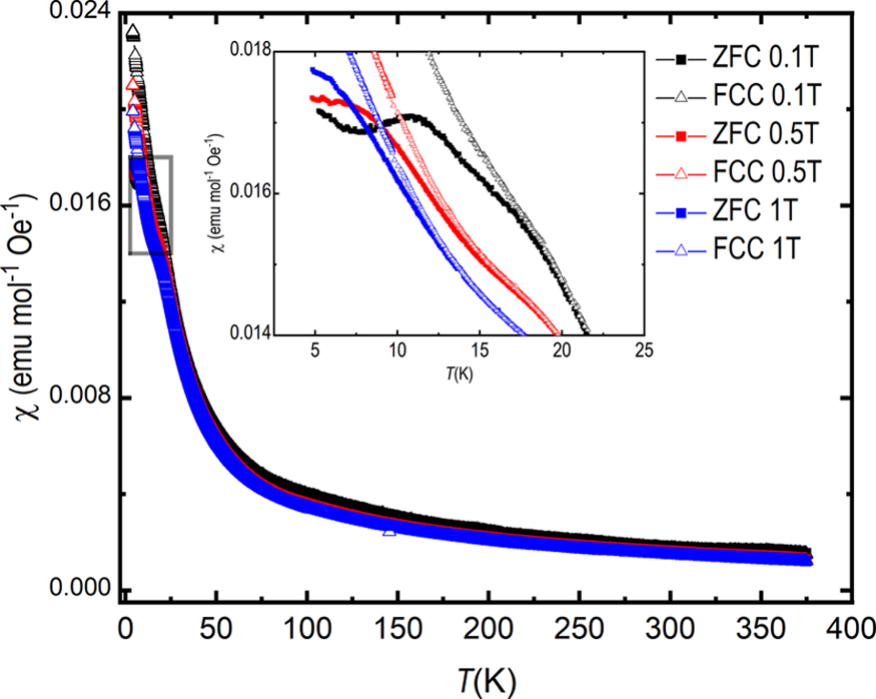
Temperature dependent magnetic susceptibility of Sr_2_VO_3_Cl at three different magnetic fields, in field-cooled cooling (FCC) and zero field-cooled (ZFC) mode. The inset displays the low-temperature range highlighting the splitting between ZFC and FCC curves. The measured FCW curves, which were identical to the FCC curves, are omitted for clarity.

According to the magnetic susceptibility measurements, the title compound is paramagnetic down to at least 35 K as the signal increases when decreasing the temperature, following the Curie–Weiss law (Figure 4). However, at low temperatures, the magnetic behavior changes: zero-field cooled (ZFC) and field-cooled cooling (FCC)/field-cooled warming (FCW) data are different. The temperature where the curves separate depends on the field strength: for 0.1 T, ZFC and FCC/FCW curves separate at ∼18 K as compared to ∼12 K for 1 T.

**Figure 5:**
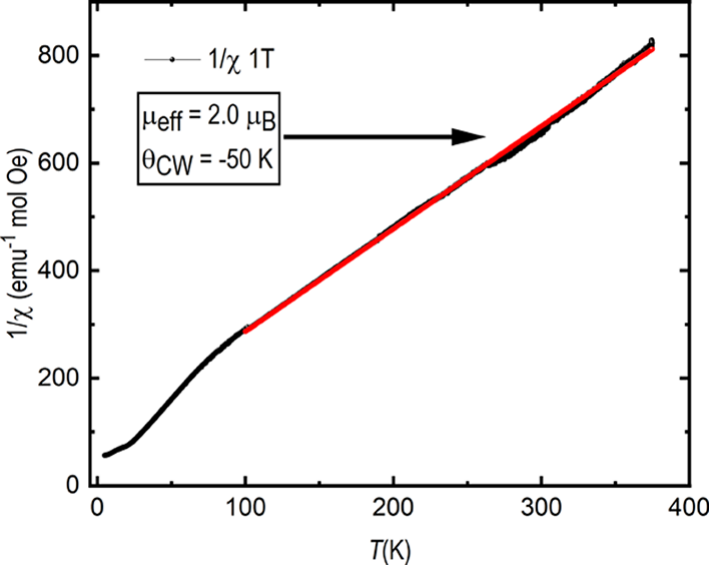
Temperature dependent inverse magnetic susceptibility of Sr_2_VO_3_Cl taken at a field of 1 T. The Curie–Weiss fit was done from 100 to 375 K as indicated by the red line; for details see text.

As observed in Figure 5, representing a 1/χ *versus T* plot, the high-temperature paramagnetic range can be approximated with a straight line. From a Curie–Weiss approximation, the effective magnetic moment μ_eff_ = 2.0 μ_B_ V^–1^ was determined. A diamagnetic correction was not performed because its influence on the paramagnetic signal is smaller than measurement errors. This is different from the expected magnetic moment for an *S* = 1 ion, such as the spin-only V^3+^ ion: . However, if an orbital moment would be active on the vanadium ion, it would oppose the spin moment, thus explaining, in part, the observation. The Curie–Weiss temperature was determined to be θ_*CW*_ = – 50 K, indicating dominant antiferromagnetic spin–spin interactions. This is in accordance with the almost straight super-exchange paths between vanadium and oxygen.

**Figure 6:**
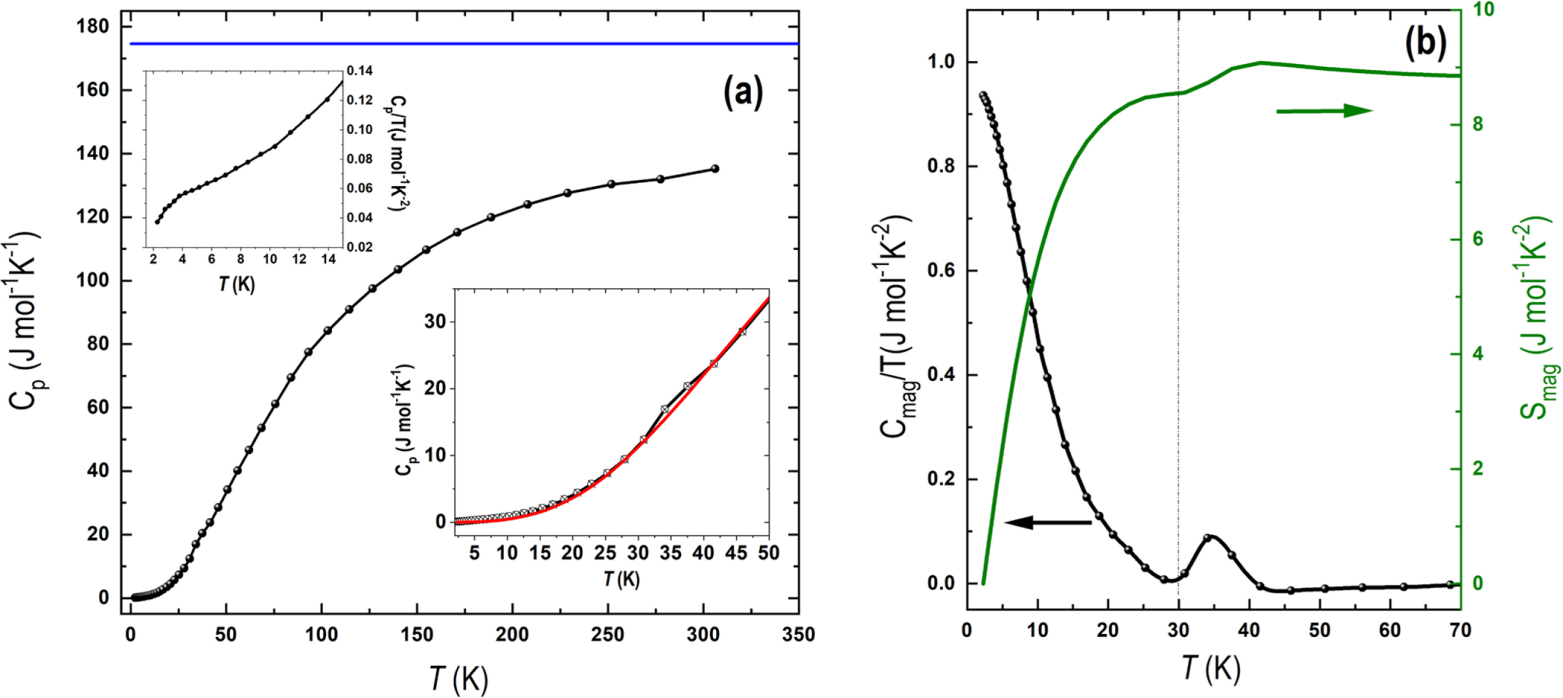
(a) Zero-field specific heat of Sr_2_VO_3_Cl. The Dulong–Petit limit 3R*n* is indicated by the blue line (*n* = 7). Upper inset: zoom into the C_p_/*T* data showing a small anomaly at low temperature. Lower inset: specific heat in the temperature range of interest, the red line is the lattice contribution estimated from fitting the experimental data with a Debye model (see text for details). (b) Magnetic contribution to the specific heat plotted as *C*_p,mag_/*T* vs *T* (left axis) and the magnetic entropy (right axis).

The specific-heat data (shown in Figure 6a) indicate that the phonon contribution dominate in the high-temperature range. As the measurement was performed on a cool-pressed polycrystalline sample, the coupling to the sample holder was not optimal, but always above roughly 80%. This could in part explain the difference between the *C*_p_ and the Dulong–Petit limit close to room temperature. Interestingly, extra contributions to the specific heat appear below 5 and around 35 K, as shown in the insets of Figure 6a.

In order to obtain the magnetic contribution to the specific heat, the lattice contribution was estimated by fitting the experimental *C*_p_ data, excluding points in the ranges *T* < 8 K and 25 K < *T* < 50 K, where significant features were observed. The fit was performed using the following Debye model:
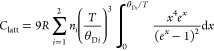
where *R* is the universal gas constant, the index *i* sums over two different Debye temperatures Θ_D1_ and Θ_D2_, each one representing a part of the total unit cell formed by *n* = 7 atoms (*n*_1_ = 3 and *n*_2_ = 4). The total estimated lattice contribution (shown by the red line in the lower inset of Figure 6a) was obtained by extrapolating the fit function to the temperature range 2 K < *T* < 70 K.

After subtracting the lattice contribution from the experimental *C*_p_ data, the magnetic contribution to the specific heat was obtained, which is plotted as *C*_mag_/*T* as a function of temperature in Figure 6b (left axis). A clear peak-like feature is observed between 30 and 40 K. The magnetic entropy (*S*_mag_) was evaluated by integrating *C*_mag_/*T* as shown in Figure 6b (right axis). An entropy release of approximately 8.8 J mol^–1^ K^–1^ at 70 K is, in principle, close to the expected for a *S* = 1 ground state, (*R* ln(2*S* + 1)) = 9.1 J mol^–1^ K^–1^. It is worth noticing that due to the fit approximations, the extracted magnetic contribution to the specific heat has a semiquantitative character only. Nevertheless, this does not alter the general behavior observed: as indicated by the vertical line in Figure 6b at *T* = 30 K, most of the entropy is not associated with the peak-like feature around 35 K, ruling it out as the signature of magnetic long-range ordering. Instead, roughly 96% of the entropy release takes place for temperatures *T* < 30 K, where there is no evidence of a sharp λ peak, but only a small anomaly for *T* < 5 K as shown in the upper inset of Figure 6a. The possible nature of such behavior is discussed in the following sections.

### ss-NMR

**Figure 7:**
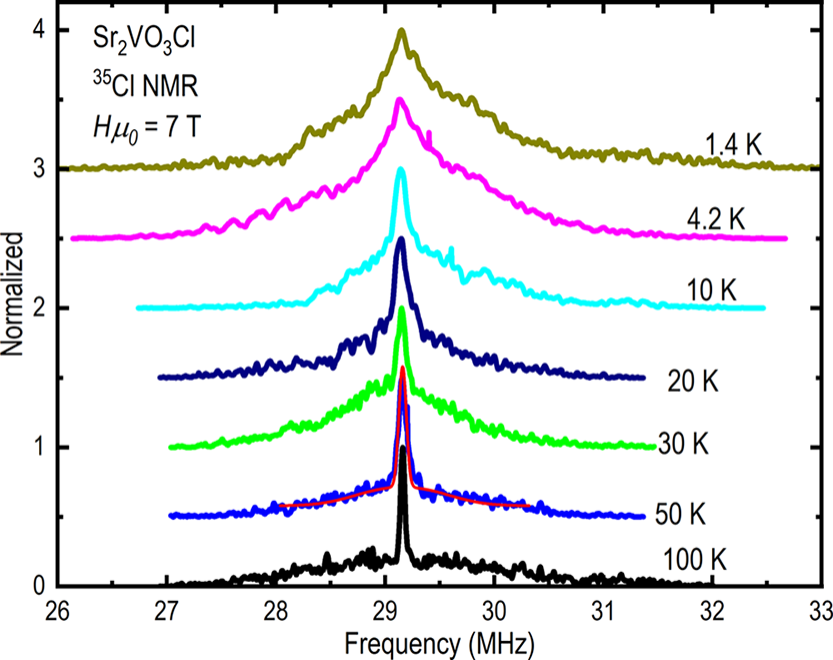
^35^Cl frequency sweep NMR spectra measured at 7 T for various temperatures. The red solid line at 50 K represents a simulation of NMR powder spectra with a quadrupole splitting of 0.02 MHz and quadrupole broadening of about 0.6 MHz.

^35^Cl NMR is used as a local probe to check for the presence of possible magnetic ordering in Sr_2_VO_3_Cl. ^35^Cl is a spin-3/2 nucleus for which one would expect a powder NMR spectrum with a central line (1/2 ↔ −1/2 transition) together with quadrupole singularities on both sides (corresponding to the −3/2 ↔ −1/2 and 3/2 ↔ 1/2 transitions). Figure 7 shows the frequency sweep ^35^Cl NMR spectra measured at *H* = 7 T. At high temperatures, the NMR spectra consist of a single and narrow central line with a broad background. This can be qualitatively described by a powder distribution with a small quadrupole splitting of about 0.02 MHz and a large quadrupole broadening of about 0.6 MHz (see the fit at 50 K). The NMR lines broaden monotonically as the temperature is lowered, and no evidence of magnetic long-range ordering is observed, while the line broadening below 4.2 K is likely due to spin freezing at low temperatures.

## Discussion

**Figure 8:**
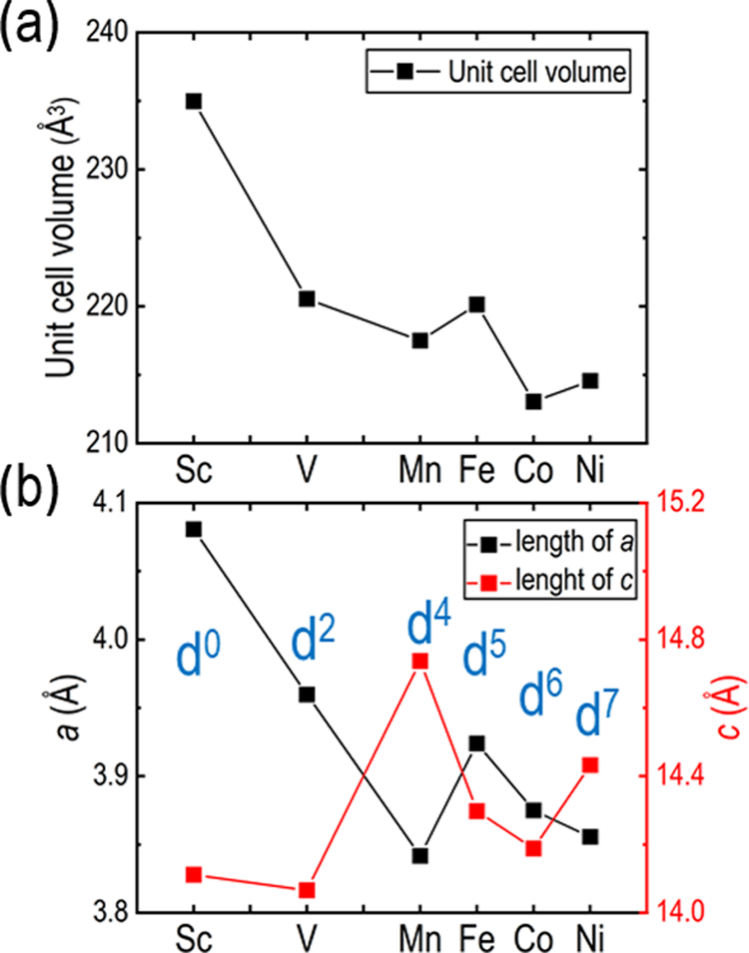
(a) Comparison of the unit cell volume *vs* 3d-metal and (b) the length of the unit cell vectors *a* (black) and *c* (red) vs 3d-metal. The number of d-electrons are indicated in blue. The comparison is made using Sc,^5^ V, Mn,^6^ Fe,^24^ Co^8^ (determined at 200 K), and Ni.^9^ All errors in the unit cell parameters are smaller than the markers.

As all known compositions in the Sr_2_*Tm*O_3_Cl series are iso-structural, changes in unit cell parameters can be used to estimate local properties, such as the electronic configuration of the transition metal. The unit cell volume of the Sr_2_*Tm*O_3_Cl members (Figure 8a) scales relatively well with increasing effective nuclear charge going from Sc to Ni. For the unit cell parameter *c* (Figure 8b), a sudden change in the trend is observed for both Mn and Ni. As V(III) is d^2^, and the coordination is quasi octahedral (Schönflies symmetry, *C*_4*v*_), the electrons will occupy *B*_2*g*_ and *E_g_* orbitals (corresponding to the *t*_2*g*_ orbitals in an octahedral field).^19^ Fe(III) (d^5^) and Co(III) (d^6^) have been determined in previous works to possess high-spin (HS) configurations^20,21^ with the electronic configurations being *B*_2*g*_^1^*E*_*g*_^2^*B*_1*g*_^1^*A*_1*g*_^1^ and *B*_2*g*_^2^*E*_*g*_^2^*B*_1*g*_^1^*A*_1*g*_^1^, respectively. In contrast, Mn(III) (d^4^) and Ni(III) (d^7^) have only one electron occupying the *B*_1*g*_ orbital with their respective electronic configurations *B*_2*g*_^1^*E*_*g*_^2^*B*_1*g*_^1^ and *B*_2*g*_^2^*E*_*g*_^4^*B*_1*g*_^1^.^22,23^ The single electron in the *B*_1*g*_ orbital could result in Jahn–Teller distortions and therefore explain the suddenly elongated *c* unit cell vector in comparison with the other homologues.

On further examining the magnetic data of Sr_2_*Tm*O_3_Cl (Figure 5), a difference between ZFC and FC curves is observed. The usual interpretation of this observation is the existence of magnetic domains. Nevertheless, long-range magnetic ordering is excluded considering that typical signs of second-order transitions are absent in the specific heat. On the other hand, fluctuating short-range AFM correlations, supplied by the advantageous super-exchange vanadium–oxygen paths, cannot explain the ZFC/FC splitting in the magnetization curves. Instead, based on the ss-NMR data, a freezing of spin-clusters (or soft domains) at lower temperatures is a tentative explanation that agrees well with a magnetic field dependent freezing temperature. This scenario is supported by the magnetic entropy calculation, where it is observed that a large part of the entropy is released at low temperature. Alternatively, the anomaly observed in the specific heat below 5 K could be associated to a two-level Schottky anomaly. As already reported,^19^ the quasi-octahedral coordination of vanadium has a C_4*v*_ symmetry that leads to d-orbital splitting where the d_*xz*_ and d_*yz*_ are degenerate but different from d_*xy*_. These discrete energy levels could then dominate the behavior of the system when thermal excitation is comparable to the energy spacing between d orbitals. The probability of populating the upper level via thermal excitations can manifest itself as a broad peak in the specific heat. Systematic measurements under several applied magnetic fields would be necessary to confirm a probable Schottky character of the anomaly, which is, however, beyond the scope of this work.

Experimentally determined Weiss constants, Néel temperatures, *Tm*–O–*Tm* angles, and *Tm*–O distances observed in the Sr_2_*Tm*O_3_Cl (*Tm* = Sc, Mn, Fe, Co, Ni) analogues are shown in Table 2.

**Table 2:** Comparison of the Weiss Constants, Néel Temperatures, *Tm*-O_eq_-*Tm* Angles, and *Tm*-O_eq_ Distances for the Sr_2_*Tm*O_3_Cl (*Tm* = Sc, V, Mn, Fe, Co, Ni) Analogues^a^

compound	*T*_N_/θ (K)	*Tm*–O_eq_–*Tm* angle (°)	*Tm*–O_eq_ distance (Å)	references
Sr_2_ScO_3_Cl	-	162.2(2)	2.0652(5)	(5)
Sr_2_VO_3_Cl	-/–50	161.7(5)	2.005(2)	this work
Sr_2_MnO_3_Cl	80(3)/–284(5)	163.2(2)	1.9417(5)	(6,22)
Sr_2_FeO_3_Cl	∼330(5)/-	160.43(1)	1.9908(2)	(20,24)
Sr_2_CoO_3_Cl	330(5)/-	160.0(4)	1.969(4)	(8,21)
Sr_2_NiO_3_Cl	33/24.2	163.6(3)	1.9477(9)	(9)

a The angles and distances are between the *Tm*s and the equatorial oxygen atoms.

When comparing the magnetic interaction strengths for the different homologues, it is obvious that the transition metal has a great influence on the magnetic behavior of the RP oxide chlorides: the Weiss constants range from negative to slightly positive. A special case is seen for the Ni analogue where a positive Weiss constant is observed, although its ground state is indeed AFM ordered.^9^ The fact that Co and Fe analogues are already AFM ordered at room temperature can be explained by their relatively larger magnetic moment of high-spin d^5^ and d^6^ ions. However, Mn (HS d^4^) and Co (HS d^6^) have the same *S* moment, but differ in the number of electrons in the *B*_1*g*_ and *A*_1*g*_ orbitals; Co has one electron in all but the doubly occupied *B*_2*g*_ orbital, making it more capable of being involved in relatively stronger super-exchange interactions. As expected, due to the different spin sizes, the homologues with *Tm* = V^3+^ (d^2^) and *Tm* = Mn^3+^ (d^4^) have very different mean super-exchange interaction strengths. No obvious trends are observed between the *Tm*–O_eq_–*Tm* angles/*Tm*–O_eq_ distances and the magnetic interactions.

## Conclusions

A new Sr_2_*Tm*O_3_Cl (*Tm* = Sc, Mn, Fe, Co, Ni) analogue with *Tm* = V^3+^ has been discovered, as observed by X-ray diffraction data and elemental analysis. Thermodynamic and ^35^Cl NMR measurements suggest a high-temperature paramagnetic state with spin freezing at low temperatures. A comparison among the structural homologues reveals that the possible transition metal electronic configuration has a strong influence on the unit cell parameters. The choice of *Tm* also strongly influences the magnetic interaction strengths and the magnetic ground state in a nontrivial way. Obviously, the higher homologues of the RP phases with chlorine replacing oxygen are a promising area for future research.

## Supporting Information Available

CIF file from JANA2006 containing the results from the here-used Rietveld refinement of powder X-ray data (CIF)

## Supplementary Material

ao3c01151_si_001.cif
